# Molecular Characterization of Lipopolysaccharide Binding to Human **α**-1-Acid Glycoprotein

**DOI:** 10.1155/2012/475153

**Published:** 2012-12-20

**Authors:** Johnny X. Huang, Mohammad A. K. Azad, Elizabeth Yuriev, Mark A. Baker, Roger L. Nation, Jian Li, Matthew A. Cooper, Tony Velkov

**Affiliations:** ^1^Institute for Molecular Bioscience, The University of Queensland, 306 Carmody Road, St. Lucia, QLD 4072, Australia; ^2^Drug Development and Innovation, Drug Delivery, Disposition and Dynamics, Monash Institute of Pharmaceutical Sciences, Monash University, 381 Royal Parade, Parkville, VIC 3052, Australia; ^3^Medicinal Chemistry, Monash Institute of Pharmaceutical Sciences, Monash University, 381 Royal Parade, Parkville, VIC 3052, Australia; ^4^Priority Research Centre in Reproductive Science, School of Environmental and Life Sciences, University of Newcastle, Callaghan, NSW 2308, Australia

## Abstract

The ability of AGP to bind circulating lipopolysaccharide (LPS) in plasma is believed to help reduce the proinflammatory effect of bacterial lipid A molecules. Here, for the first time we have characterized human AGP binding characteristics of the LPS from a number of pathogenic Gram-negative bacteria: *Escherichia coli*, *Salmonella typhimurium*, *Klebsiella pneumonia*, *Pseudomonas aeruginosa*, and *Serratia marcescens*. The binding affinity and structure activity relationships (SAR) of the AGP-LPS interactions were characterized by surface plasma resonance (SPR). In order to dissect the contribution of the lipid A, core oligosaccharide and *O*-antigen polysaccharide components of LPS, the AGP binding affinity of LPS from smooth strains, were compared to lipid A, Kdo2-lipid A, R_a_, R_d_, and R_e_ rough LPS mutants. The SAR analysis enabled by the binding data suggested that, in addition to the important role played by the lipid A and core components of LPS, it is predominately the unique species- and strain-specific carbohydrate structure of the *O*-antigen polysaccharide that largely determines the binding affinity for AGP. Together, these data are consistent with the role of AGP in the binding and transport of LPS in plasma during acute-phase inflammatory responses to invading Gram-negative bacteria.

## 1. Introduction

The human body is continuously challenged by infectious microorganisms. Accordingly, it has evolved numerous mechanisms for the early recognition and efficient elimination of viable microbes and their remnants [1, 2]. For defense against invading Gram-negative bacteria, the recognition of bacterial cellular components such as LPS by the innate immune system is an important event for induction of the inflammatory immune response, which is responsible for targeting the invading microorganisms and for elimination and clearance of highly endotoxic LPS [1–4].

**Figure 1:**
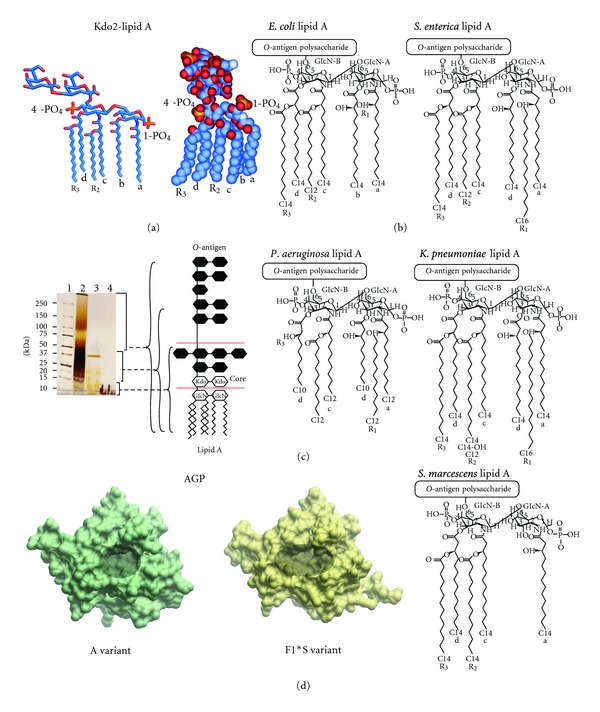
(a) Structure of Kdo2-lipid A shown in stick (left-hand panel) and CPK (right-hand panel) representation. (b) Chemical structures of the lipid A component of the LPS samples used in this study [41–43]. (c) 4–20% SDS-PAGE separation of the LPS samples used in this study. The well samples contained 30 *μ*g of each LPS sample. The gel was stained by the silver staining technique used for LPS [44]. The structural organization of LPS is shown schematically in the right-hand panel. Lane 1: molecular weight; Lane 2: *E. coli* O111:B4 LPS; Lane 3: *E. coli* EH100 (R_a_) LPS: Lane 4. *E. coli* F583 (R_d_) LPS. (d) Surface representation of the A (PDB ID: 3APU) and F1*S (PDB ID: 3KQ0) variants of human AGP. The ligand binding pocket of each variant is highlighted by the shaded area.

LPS is present only in the outer leaflet of the outer membrane (OM) in Gram-negative bacteria [5–7]. Structurally, LPS of enterobacteria consists of three components: (1) lipid A, a disaccharide acylated with fatty acid chains which is the toxic component of LPS; (2) the core region, a nonrepetitive oligosaccharide (~9 sugars in length) which can be subdivided into the inner and outer parts; (3) *O*-antigen, a serogroup-specific polysaccharide of repetitive oligosaccharide units (Figure 1(c); Table 1) [5–7]. LPS mediates a range of pathophysiological processes, more specifically, it is the lipid A component that is responsible for inducing the immunopathogenic processes that can lead to endotoxemia-associated high mortality [3, 8, 9]. Lipid A is bound by the toll-like receptor 4 (TLR4) expressed on the membrane of macrophages and neutrophils [2–4]. Activation of TLR4 by LPS is also dependent on interactions with an additional cell surface co-receptor MD-2 [2–4]. Moreover, CD14 and LPS-binding protein (LBP) are known to facilitate the presentation of LPS to MD-2 [2–4]. Once activated, the LPS-TLR4 complex stimulates signal transduction pathways that initiate the production of inflammatory cytokines, chemokines and, after hepatocyte activation, acute-phase proteins such as *α*_1_-acid glycoprotein (AGP) that are central components of the inflammatory immune response to the invading microbe [3, 4]. The LPS-induced overstimulation of the immune system can lead to the excessive release of these endogenous inflammatory mediators, resulting in multi-organ failure, septic shock syndrome and even death [2, 3, 8].

Human AGP exists as three genetic variants, the A variant, the F1 and S variants [10–15]. The expression of human AGP is under the control of two adjacent genes ORM1 (*syn.* AAG-A) and ORM2 (*syn.* AAG-B/B′), situated on chromosome 9 [11, 12]. The more active of the two, ORM1, that is induced during acute phase reactions, encodes the F1 and S variants, and ORM2 encodes the A variant [10–15]. The precursor product of the ORM1 gene is a 201 amino acid polypeptide with an 18 residue *N*-terminal secretory peptide that is cleaved [10–15]. The F1 and S variants, encoded by two alleles of the ORM1 gene differ only in a single amino acid codon (Gln20→Arg), and hereon in shall be referred to collectively as the F1*S variant. The ORM2 gene displays 22 base substitutions, which translates into 21 amino acid substitutions between the F1*S and A protein variants [10–12]. The resolution of the crystallographic structures of the human F1*S and A AGP variants revealed that the binding cavity of each variant is sub-divided into lobes [16, 17]. This intricate cavity organization suggests the AGP-drug binding site consists of partially overlapping sub-sites as opposed to the existence of distinct binding sites for acidic, basic and non-polar ligands (Figure 1(d)) [16–18]. On the primary level, AGP is composed of a single polypeptide chain of 183 amino acids [15, 19, 20]. The polypeptide component only contributes about a half of its total molecular mass of approximately 41 kDa, the rest of its mass derives from the five *N*-linked sialyl-glycans which confer AGP with a net negative charge at physiological pH [21–23]. These features also render AGP very soluble and acidic (pI ~ 2.8–3.8) [15, 19, 20]. 

In healthy individuals, the basal plasma concentration of AGP is approximately 0.7–1.0 g/L making it one of the predominant plasma proteins. The plasma AGP concentrations can fluctuate widely between health and disease, in diseased states such as sepsis, AGP levels can increase up to 5-fold [15, 35–37]. Therefore, the effect of AGP binding on the activity of highly bound substance scan be significant during acute-phase reactions. Although AGP is an abundant plasma protein, its true physiological significance remains enigmatic. However, the time course of AGP production during acute-phase responses together with its high avidity for both exogenous and endogenous inflammatory mediators supports an immune-modulatory and/or transporter activity [19, 20, 38, 39]. AGP has been implicated in being part of the physiological response to a variety of insults such as major trauma, tissue necrosis, microbial infection, and exacerbations of inflammatory diseases [19]. Several lines of evidence suggest that one of the potential physiologically functions of AGP is to bind LPS and exert immune-modulatory and/or transporter functions in relation to LPS during the acute-phase inflammatory response to Gram-negative bacterial infection: (1) AGP was demonstrated to bind directly with* E. coli* O111:B4 LPS using dynamic light scattering particle sizing, particle mobility and flow microcalorimetry techniques [39]. (2) AGP agglutinated LPS impregnated rabbit red blood cells [39]. (3) In a meningococcal endotoxemia mouse model, the intraperitoneal administration of 8 mg of bovine AGP 2 h prior to LPS challenge was shown to protect against sepsis [39]. (4) Intraperitoneal administration of 10 mg AGP, 2 h prior to a lethal challenge of *K. pneumoniae* increased the survival of mice [40]. (5) Transgenic over expression of rat AGP protected mice from a lethal challenge of *K. pneumonia *[40]. 

Notwithstanding the noted *in vitro*-*in vivo* correlations, these previous studies have not addressed any structural details of the LPS-AGP interaction. A better understanding of the molecular mechanisms that drive the interaction between LPS and central components of the host inflammatory response to infection (such as LPS-AGP complexation) is needed to enable the development of new treatment strategies for severe sepsis. This study is the first to utilize SPR and fluorometric binding assays together with molecular modeling to characterize the SAR that drive the binding of LPS to human AGP. 

## 2. Materials and Methods

### 2.1. Materials

Human AGP, fluorescein isothiocyanate- (FITC) labeled *Escherichia coli *O111:B4LPS, and LPS from *E. coli *O111:B4, *E. coli* O127:B8 (ATCC 12740), *E. coli* EH-100 (R_a_ mutant) LPS, *E. coli *F-583 (R_d_ mutant) LPS, *Klebsiella pneumonia *(ATCC 15380) LPS, *Pseudomonas aeruginosa *serotype 10 LPS, *Salmonella typhimurium *(ATCC 7823) LPS, *Salmonella typhimurium *strain TV119 (R_a_ mutant) LPS, *Salmonella typhimurium *strain SL1181 (R_e_ mutant) LPS, *Serratia marcescens* (ATCC 21639) LPS, and diphosphoryl lipid A from *E. coli *were obtained from Sigma-Aldrich (Sydney, NSW, Australia). Kdo2-lipid A was obtained from Avanti Polar Lipids (Alabama, USA). All other reagents were of the highest grade commercially available.

### 2.2. Fluorometric Assay of FITC-*E. Coli* O111:B4 LPS Binding to AGP

The binding of FITC-LPS to AGP was measured by titrating a 1 mL solution of FITC-LPS (1.5 *μ*M) in a quartz cuvette with aliquots of AGP (20–50 *μ*M). Fluorescence was measured using a Cary Eclipse Fluorescence spectrophotometer (Varian, Mulgrave, VIC, Australia) set at an excitation wavelength specific for the FITC fluorophore (Ex*λ* 494 nm). Slit widths were set to 5 nm for both the excitation and emission monochromators. The emission spectrum was collected in the 500→650 nm range. 

### 2.3. Surface Plasmon Resonance (SPR) Assay of LPS Binding to Human AGP

SPR experiments were performed using a Biacore T200 instrument. AGP was immobilized on a CM5 sensor chip surface using the surface thiol coupling method following manufacturer's instructions (GE Health Care, Melbourne, Australia). In brief, 0.5 mg AGP was dissolved in 0.5 mL of 0.1 M morpholino-ethane sulfonic acid (MES) buffer pH 5.0. Then 250 *μ*L of 15 mg/mL 2-(2-Pyridinyldithil)ethaneamine hydrochloride (PDEA) and 25 *μ*L of 0.4 M 1-ethyl-3-(3-dimethylaminopropyl)carbodiimide hydrochloride (EDC) were added and incubated at room temperature for 10 min. After the reaction, excess reagents were removed using a PD10 desalting column. The PDEA-modified AGP was then used for thiol coupling. After the introduction and reduction of disulfide group on the CM5 chip, PDEA-modified AGP was injected across the surface at a flow rate of 10 *μ*L/min for 7 min. The immobilization level of AGP was 5000 RU. Solutions of LPS samples were prepared in either HBS-EP (10 mM HEPES pH 7.4, 150 mM NaCl, 3 mM EDTA, and 0.005% (v/v) surfactant P20) or HBS-P (10 mM HEPES pH 7.4, 150 mM NaCl, and 0.005% (v/v) surfactant P20) running buffer. The LPS concentration range used in SPR experiment was 10 to 3000 *μ*g/mL, in 3-fold dilutions. LPS samples were injected across two flow cells on a CM5 chip at a flow rate of 30 *μ*L/min. Flow cell 2 was immobilized with AGP; whereas flow cell 1 was deactivated and served as the blank reference surface. All SPR assays were repeated at least 3 times at 25°C. Association period was set up for 30 sec followed by 60 sec dissociation. Binding responses were recorded 10 sec before the end of association period. The reference flow cell response was subtracted from all data used in the analysis. 

In the case of lipid A and Kdo2-lipid A binding, a L1 sensor chip was used (GE Health Care, Melbourne Australia). Small unilamellar vesicles (SUVs) were prepared in PBS by sonication and extrusion. Lipids (DMPC, lipid A, and Kdo2-lipid A) were dissolved in ethanol-free chloroform in 25 mL round-bottom flasks. 10% (mol/mol) of lipid A or Kdo2-lipid A was added into DMPC solution to make 10% lipid A/Kdo2-lipid A-DMPC mixtures, which were then deposited as a thin film by removal of the solvent (chloroform) under reduced pressure on a rotary evaporator and dried under high vacuum for at least 2 hours. PBS was then added into each flask to give a 1 mM suspension, which was sonicated 5 min for 5 times. The suspension was passed 17 times through a 50 nm polycarbonate filter in an Avestin Lipofast Basic extrusion apparatus to give a translucent solution of vesicles, which should possess a mean diameter of 50 nm. The SUVs were then injected into the flow cells of the L1 sensor chip for 2000 sec at a low flow rate of 2 *μ*L/min to form a bilayer membrane model on the chip surface. Then, a series of AGP solutions was injected across the flow cells at a flow rate of 30 *μ*L/min, having an injection phase of 180 s and a dissociation phase of 300 s. A regeneration step was added before and after every cycle using 40 mM octyl-glucoside, which cleans the chip surface for a new cycle. Pure DMPC bilayer was applied as a reference and all data used in analysis were reference-subtracted. 

### 2.4. 3-Deoxy-d-manno-oct-2-ulopyranosonic Acid (Kdo) Assay

The molar concentration of Kdo in the LPS samples was measured following the purpald assay as previously described in detail [45]. 

### 2.5. Cytotoxicity Assay

HEK293 cell line was purchased from American Type Culture Collection (ATCC). The cells were cultured in DMEM (Invitrogen, Australia) containing 10% fetal bovine serum at 37°C, 5% CO_2_. Serum-free media were used in cytotoxicity assays. Cytotoxicity of LPS to HEK293 cells was determined using Alamar Blue cell viability reagent (Invitrogen, Australia). In brief, HEK293 cells were seeded as 2 × 10^4^ cells per well in a clear 96-well plate and incubated for 24 hr at 37°C, 5% CO_2_. Then the media were replaced with serum-free media, which contained AGP and LPS (100 *μ*g/mL). After a 24 hr incubation, 10 *μ*L of Alamar Blue reagent were added per well and incubated at 37°C for 1 hr. Then the fluorescence intensity was read using Polar star Omega with excitation/emission wavelengths of 560/590.

### 2.6. Molecular Modeling of the Kdo2 Lipid A-AGP Complex

The crystallographic coordinates of the F1*S variant of *apo*-human AGP were retrieved from the protein data bank (PDB ID: 3KQ0) [17]. The tetraantennary *N*-glycans characteristic for human AGP [19, 22, 46] were added to the crystallographic AGP structure using the GlyProt server (http://www.glycosciences.de/modeling/glyprot/php/main.php) [47, 48]. A docking model of Kdo2-lipid A in complex with the F1*S variant of human AGP was constructed using the Accelrys Discovery Studio V2.1 CDOCKER algorithm as per the standard protocol in the manufacturer's instructions (Accelrys, San Diego, CA, USA). 

## 3. Results

### 3.1. Fluorometric Assay of the Binding Interaction between FITC-Labeled *E. coli* O111:B4 LPS with Human AGP

**Figure 2:**
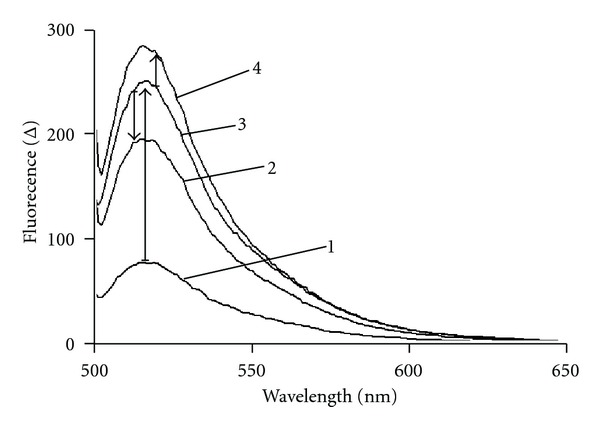
Fluorescence emission spectra of (1) FITC-labeled *E. coli* O111:B4 LPS (5 *μ*M). (2) Decrease in fluorescence observed upon addition of chlorpromazine (20 *μ*M) to the FITC-LPS:AGP complex. (3) FITC-LPS (5 *μ*M) in complex with human AGP (25 *μ*M). (4) FITC-LPS (5 *μ*M) in complex with human AGP (25 *μ*M) in the presence of EDTA (1 mM).

FITC-LPS displays a fluorescence emission maximum as a wavelength of ~515 nm (Figure 2). Fluorescence intensity was noted to increase upon titration with AGP (Figure 2). EDTA has a well-documented effect of sequestering the divalent cations that help bridge adjacent LPS molecules when they are arranged in a leaflet or aggregate structure [9]. The titration of FITC-LPS with AGP in the presence of EDTA (1 mM final concentration) produced a higher level of fluorescence emission compared to an identical titration in the absence of EDTA (Figure 2). The addition of chlorpromazine (20 *μ*M final concentration) to the FITC-LPS:AGP complex produced a decrease in fluorescence emission (Figure 2). 

### 3.2. Surface Plasmon Resonance Assay of LPS Binding to Human AGP

**Figure 3:**
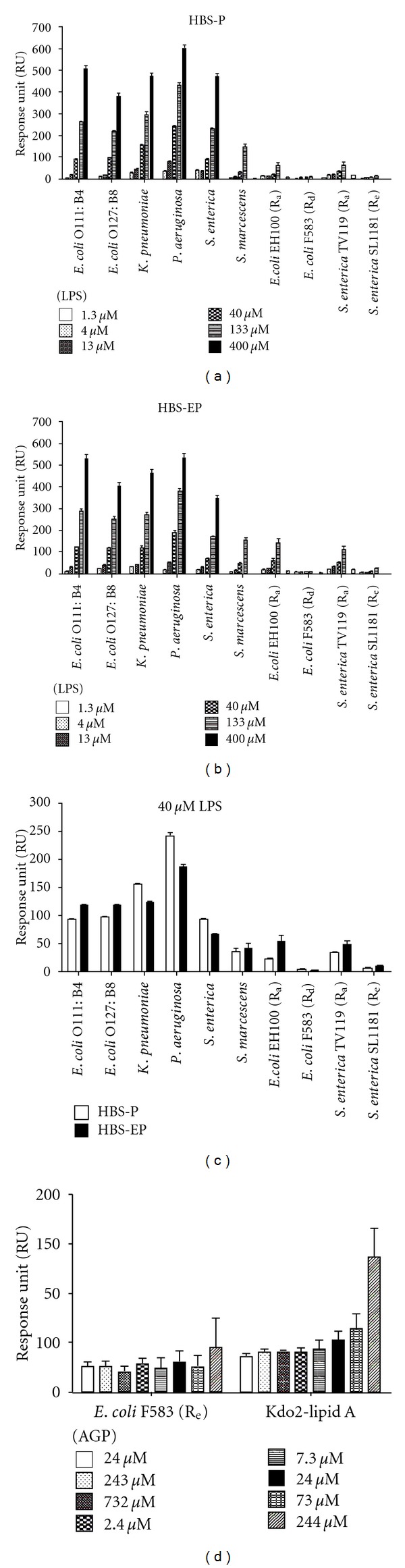
SPR assay of the binding of LPS or lipid A to AGP. (a) Binding responses over a concentration range of LPS in EDTA free (HBS-P) and (b) EDTA buffer (HBS-EP). (c) Binding responses at 40 *μ*MLPS in EDTA free (HBS-P) and EDTA buffer (HBS-EP). (d) Binding responses of AGP to DMPC bilayer incorporated 10 (mol/mol) % lipid A.

The highly variable length of the *O*-antigen polysaccharide means that LPS from different bacterial strains have different molecular weights which complicates quantitative comparisons of binding affinity. Accordingly, the molar concentration of the LPS samples was determined using the purpald Kdo assay and we standardized the concentrations of LPS in the SPR experiments. The SPR binding measurements were performed using AGP immobilized to a CM5 sensor chip surface and titrated with LPS (see Figure S1 available online at doi:10.1155/2012/475153). In order to investigate the impact of EDTA on binding, the experiments were replicated in HBS-P and HBS-EP buffers (HBS-EP buffer contains 3 mM EDTA). The binding of the LPS samples to AGP was dose-dependent (Figures 3(a) and 3(b)). The binding affinity varied between LPS isolates from different genera and between LPS isolates from different strains (Figure 3). Ranking of LPS binding to AGP was investigated at single concentrations of 40 *μ*M of LPS, which gave a rank order of affinity *P. aeruginosa *> *K. pneumonia *> *E. coli* O127:B8 > *E. coli* O111:B4 > *S. enteric *> *E. coli* EH100 (R_a_) ≥ *S. enteric* TV119 (R_a_) > *S. enteric *SL1181 (R_e_) > *S. marcescens *> *E. coli* F583 (R_d_) (Figure 3(b)). The binding of *E. coli* O111:B4, *E. coli* O127:B8, *E. coli* EH100 (R_a_), *S. enterica* TV119 (R_a_), and *S. enteric *SL1181 (R_e_) was higher in HBS-EP than in HBS-P buffer. Whereas, in the case of *K. pneumonia*, *Pseudomonas aeruginosa*, *S. enterica*, and* S. marcescens, *the presence of EDTA did not markedly affect the AGP binding levels. Because of its insolubility in the flow buffer, the binding of diphosphoryl lipid A from *E. coli *was investigated using hybrid SUVs. There was no specific AGP binding detected for diphosphoryl lipid A (data not shown). Hybrid SUV experiments were also performed with fully synthetic Kdo2-lipid A and *E. coli* F583 (R_d_) LPS (Figure 3(d)). There was no specific AGP binding detected for *E. coli* F583 (R_d_) lipid A, while as the concentrations of AGP were higher than 7 *μ*M, a dose-dependent response to Kdo2-lipid A was observed (Figure 3(d)).

### 3.3. Molecular Modeling of the Kdo2-Lipid A in Complex with F1*S AGP 

**Figure 4:**
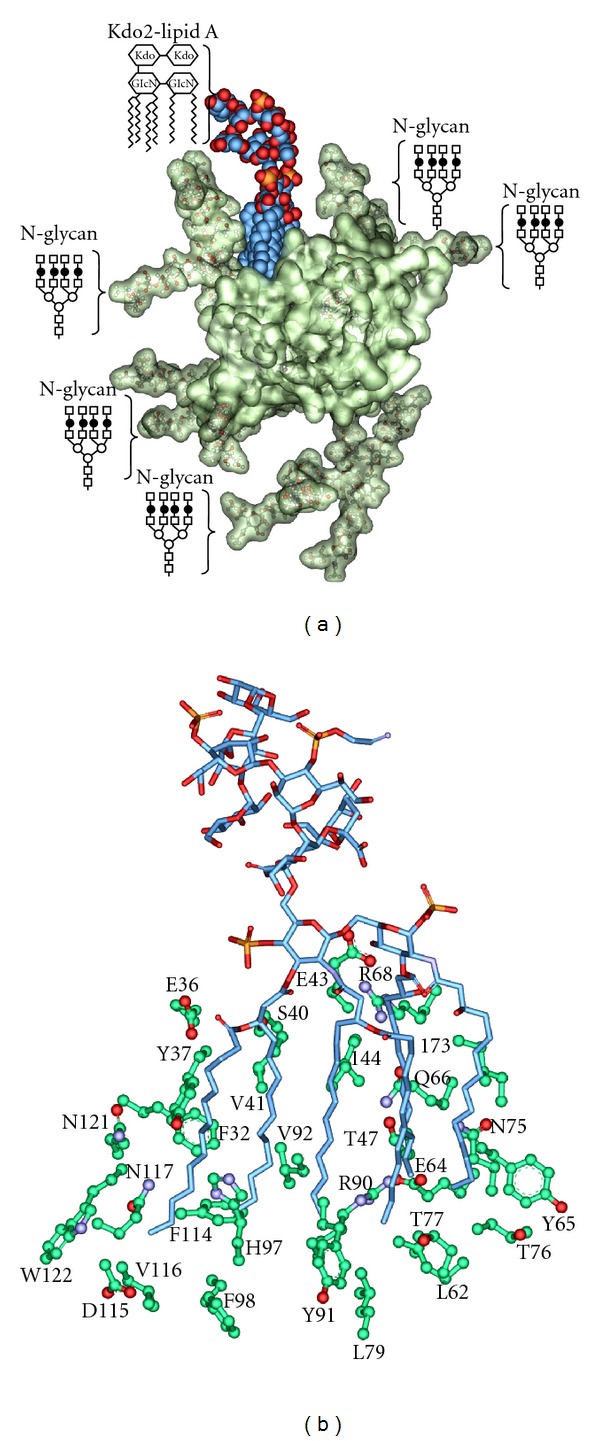
Molecular model of the Kdo2-lipid A F1*S AGP complex. (a) The AGP F1*S variant crystal structure (PDB code: 3KQ0) is shown in semitransparent surface representation, the bound Kdo2-lipid A is shown in CPK, carbon is colored blue. The Kdo2-lipid A and quaternary *N*-glycans on the AGP structure are indicated schematically (○ Man; □ GlcNAc; *⚫* d-Gal) and are shown in ball- and stick-representation on the model. (b) Interactions between the fatty acyl chains of lipid A and the side chains of the ligand binding cavity of AGP.

In an attempt to provide a structurale rational for how LPS binds to AGP we have constructed a molecular docking model of the complex using the crystallographic structure of the F1*S variant of human AGP [17] as the receptor and Kdo2-lipid A as the ligand (Figure 4(a)). The model suggests that the LPS-AGP complex is in part stabilized through contacts between the fatty acyl chains of lipid A and a set of polar and nonpolar sidechains within the ligand binding cavity of AGP (Figure 4(b)). The major contact points with the lipid A fatty acyl chains involve the AGP side chains of Phe32, Glu36, Tyr37, Val41, Ile44, Thr47, Leu62, Tyr65, Glu64, Gln66, Asn75, Thr76, Thr77, Leu79, Val92, His97, Phe98, Leu112, Phe114, Val116, Asn117, Asn121, Trp122, and the 3-carbon aliphatic segment of the Arg90 side chain (Figure 4(b)). AGP displays five *N*-linked sialyl-oligosaccharides attached to the side chain of Asn residues found at positions 15, 38, 54, 75, and 85 in its amino acid sequence [21–23]. The Asn residues at positions 15, 54, and 85 are situated at the closed end of the *β*-barrel on the opposite side of the molecule from the entrance of the ligand binding cavity and, therefore, are unlikely to interfere with ligand entry [17]. While Asn38 and 75-line the entrance to the ligand binding pocket [17, 21–23]. The five *N*-glycan sites were modeled using the reported tetra-antennary glycan structures for human AGP [19, 22, 46]. The docking model shows that the Kdo2 sugars of lipid A make polar contacts with the *N*-linked sialyl-oligosaccharides attached to Asn38 and 75 that decorate the entrance of the AGP cavity. This would suggest that the AGP-LPS complex is further stabilized through polar contacts between the core oligosaccharide and *O*-antigen with the *N*-glycan structures that decorate the surface of AGP (Figure 4(a)).

### 3.4. Protection against LPS Cytotoxicity by AGP

**Figure 5:**
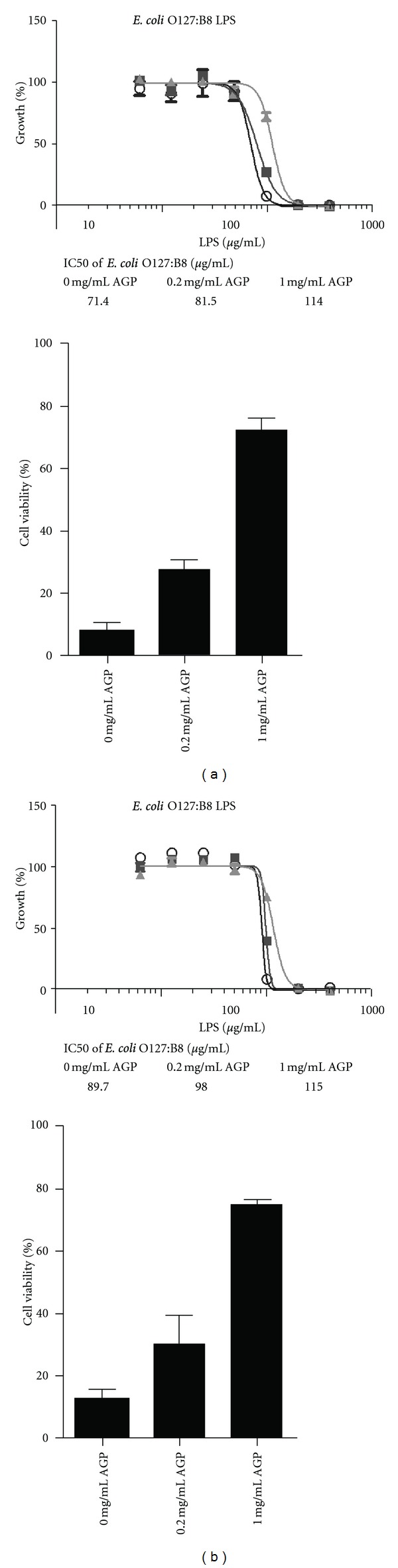
The protective effect of AGP against LPS-induced cytotoxicity in HEK293 cell culture. (a) *Top panel.* The percentage of cell growth in the presence of increasing concentrations of *E. coli* O127:B8 LPS. (○) 0 mg/mL AGP; (■) 0.2 mg/mL AGP; (▲) 1 mg/mL AGP. *Bottom panel.* The percentage of cell viability upon exposure to 100 *μ*g/mL *E. coli* O127:B8 LPS, in the presence and absence of AGP. (b) *Top panel.* The percentage of cell growth in the presence of increasing concentrations of *E. coli* O111:B4 LPS. (○) 0 mg/mL AGP; (■) 0.2 mg/mL AGP; (▲) 1 mg/mL AGP. *Bottom panel.* The percentage of cell viability upon exposure to 100 *μ*g/mL *E. coli* O111:B4 LPS, in the presence and absence of AGP. The inset table documents the IC50 values for LPS in the presence of increasing AGP levels.

In order to investigate the protective effect of AGP on mammalian cells in the present of LPS, we performed a cell viability assay using HEK293 cells. The exposure of HEK293 cells to 100 *μ*g/mL *E. coli* O111:B4 LPS induced more than 80% cell death with an IC50 value of 89.7 *μ*g/mL (Figure 5). Similar results were observed with *E. coli* O127:B8 LPS which elicited an IC50 value of 71.4 *μ*g/mL (Figure 5). The cytotoxic effect of LPS could be ameliorated in a concentration-dependent manner by AGP (Figure 5). In the presence of 1 mg/mL AGP, which is approximately equivalent to the human plasma concentration of AGP in healthy individuals (20 *μ*M) [14, 49, 50], 80% of the HEK293 cells retained viability after a 24 hr incubation with 100 *μ*g/mL *E. coli* O111:B4 LPS (Figure 5). The IC50 values for both LPS samples decreased in the presence of 1 mg/mL AGP, indicative of a protective effect (Figure 5, insets). 

## 4. Discussion

### 4.1. Structure-Activity Relationships for the LPS-AGP Interaction

In order to investigate the SAR of the LPS-AGP interaction we examined the contribution of the three main components of LPS, namely, (1) lipid A; (2) the core oligosaccharide; (3) the *O*-antigen polysaccharide. 

Bacteria that express LPS with a complete core and *O*-polysaccharide are referred to as “smooth” strains, a term originally coined by Griffith based on his observation of their colony morphology [51]. In contrast, mutant bacteria that have defects in the LPS biosynthetic pathway and produce LPS without *O*-polysaccharide and/or contain truncated core oligosaccharides are referred to as “rough” strains [6, 51]. The structural analysis of LPS from *Salmonella* rough mutants led to the differentiation of the R_a_ to R_e_ chemotypes [6]. R_a_ mutants have defects in the *O*-antigen biosynthetic pathway and display a LPS chemotype with a complete core structure minus the *O*-polysaccharide [6]. Whereas at the other extreme, R_e_ mutants display the smallest viable core structure required for growth, consisting of the Kdo2-lipid A “backbone” which lacks all of the core sugars except for the Kdo disaccharide (Figure 1(a); Table 1) [6]. The other chemotypes R_b_, R_c_, and R_d_ display core lengths intermediate to the R_a_ and R_e_ extremes [6] (Table 1). To determine the contribution of the *O*-antigen and core carbohydrate structures towards AGP binding we examined the binding of AGP with LPS isolated from R_a_, R_d_, and R_e_ mutants of *S. enterica* and *E. coli*, and in addition, fully synthetic Kdo2-lipid A. The contribution of the lipid A component for AGP binding was measured using diphosphoryl lipid A prepared from *E. coli* LPS (Figure 1(b); Table 1). 

#### 4.1.1. Lipid A

The AGP binding affinity of the lipid A component of LPS was measured using diphosphoryl *E. coli* lipid A (Figure 1(b)) embedded in SUVs. The binding of lipid A could not be measured directly due to the insolubility of lipid A in the SPR flow buffer. The SPR data indicated that AGP did not bind lipid A embedded in SUVs, suggesting that the core and *O*-antigen carbohydrate structures are indispensable for the binding of LPS to AGP. Alternatively, it is also possible that the key structural components of lipid A required for the interaction with AGP are inaccessible in the SUV arrangement.

#### 4.1.2. Core Oligosaccharide

The core polysaccharide consists of a short chain of sugars that is invariably attached to the lipid A component via a Kdo residue through a ketosidic linkage [7, 30, 31]. The core is usually conserved across members of a bacterial genus, but can be quite variable between genera of Gram-negative bacteria [7, 30, 31]. Nevertheless, this structural variability is minor in comparison with that of the *O*-antigen polysaccharide. The inner core polysaccharide of Enterobacteriaceae is composed of L-*glycero*-d-*manno *heptose (heptose) and Kdo [7, 30, 31], usually carrying charged moieties such as phosphate and phosphorylethanolamine (Table 1). The heptoses sugar occurs as either l,d-Hep or in addition to d,d-Hep as per the core of *S. marcescens *(Table 1) [7, 30, 31, 33, 34]. The outer core is composed predominantly of common hexose sugars, glucose (Glu), galactose (Gal), and *N*-acetylglucosamine (GlcNAc) (Table 1). These basal sugars are often decorated with groups such as phosphate, sulfate, ethanolamine, acetyl, amino acids, or phosphodiester-linked derivatives [7, 30, 31]. The chemical structures of core oligosaccharides from the LPS samples used in this study are been summarized in Table 1. While each of these core structures is structurally similar, there are a number of strain- and genera-specific differences in sugar composition and linkage configuration within the respective core regions. In *E. coli *LPS five distinct core structures have been characterized, termed K-12 and R1, R2, R3, R4 [7, 30, 31]. The serotype O127:B8 and EH100 R_a_ LPS possess the R2 core type, whereas the serotype O111:B4 strain possesses the R3 core type (Table 1). The SPR results did not show any marked differences in the AGP binding between O111:B4 and O127:B8 LPS suggesting the contribution of the core structure is secondary to the *O*-antigen polysaccharide (Figure 3). Two core types have been described for *S. enterica*, the test LPS from the smooth strain and from the TV119 R_a_ rough mutant both display the same core structure (Table 1) [30, 31]. The cores of the *E. coli* and *S. enterica* test strains are composed of the common hexoses, glucose (Glu), galactose (Gal), and *N*-acetylglucosamine (GlcNAc). The core regions of test LPS samples from *E. coli*, *S. enterica*, and *P. aeruginosa* are all phosphorylated at the two inner heptoses [27–31, 52]. Furthermore, the phosphate on the first heptosein these core structures is further derivatized with ethanolamine phosphate (PEtN), forming ethanolamine diphosphate [27–31, 52]. In addition, the *P. aeruginosa* core displays the l-rhamnose (Rha) and d-galactosamine (GalN) “special” sugars; 7-*O*-carbamoylation of HepII and *N*-acylation of GalN residue with l-alanine [27–29, 52]. As per *S. enterica*, thus far, only two core types have been described for *K. pneumonia *[25, 26]. In addition to the common hexoses, the core of the LPS from the *K. pneumonia* serotype O2 test strain contains the Kdo and galacturonic acid (GalA) “special” sugars (Table 1) [25, 26]. Unlike the core oligosaccharides of *E. coli*, *S. enterica* and *P. aeruginosa *are rich in negative charges which arise from the phosphate substitution of the Hep sugars [27–31, 33, 52]; one noticeable feature of the *K. pneumonia *and* S. marcescens s*cores is the absence of phosphate residues. Instead the carboxylic acid groups of the Kdo and GalA residues provide the negative charges in their core oligosaccharides [25, 26, 33, 34]. Despite these structural differences across the core structures of the LPS test samples, it is difficult to differentiate the contribution of the individual core oligosaccharides in the smooth LPS strains due to the presence of the highly variable *O*-antigen in these structures. However, the SPR results from the titrations with the *E. coli* and *S. enteric *R_a_, R_d_, and R_e_ rough mutant LPS samples (which lack the *O*-antigen component), suggest that the full-length core oligosaccharide structure is required for avid binding to AGP (Figure 3). The AGP binding affinity was much weaker with the R_d_ and R_e_ rough LPS samples (in which the outer core sugars are absent) compared to the R_a_ samples, which displays the full-length core oligosaccharide structure (Figure 3; Table 1). Similarly, the AGP binding of the fully synthetic Kdo2-lipid A structure (homologous with the R_e_ LPS structure) was found to be very weak (Figure 3).

#### 4.1.3. *O*-Antigen Polysaccharide

The SPR experiments revealed that the binding of AGP to LPS is predominantly dependent on its interaction with the *O*-antigen polysaccharide region of LPS, which appears to be more important than the interactions with the core and lipid A regions. The *O*-antigen is a large polymer attached to the outer core that extends into the environment and represents the most heterogenous region of the LPS molecule [24]. The *O*-antigen is made up of repeats of the same oligosaccharide unit, generally consisting of two to six sugar residues (Table 1) [24]. The *O*-antigen polysaccharide chain length is highly variable among bacterial strains and can range up to 10–40 repeating units [24]. So it follows, the LPS from a given bacteria strain is a heterogeneous mixture of LPS molecules differing in the length of the *O*-antigen polysaccharide. The heterogeneity in the number and distribution of *O*-antigen repeats of LPS samples are seen as a ladder of high molecular weight bands following SDS-PAGE and silver staining (Figure 1(c)). In addition to the highly variable chain length, there are variations in the sugar content, at least 20 different types of sugar are found in the *O*-antigens of Enterobacteriaceae [24]. This inherent variability gives rise to the diversity of antigenic types and forms the foundation of *O*-serotyping for species of Gram-negative bacteria [24]. For example, *Salmonella spp.* display over 1000 distinct *O*-antigens [24]. Moreover, the heterogeneity within the *O*-antigen structure also arises from the anomeric linkages between sugars and from nonstoichiometric tailoring modifications to the sugar moieties, such as the addition of phosphate, acetyl, or methyl groups [24]. The heterogeneity of the *O*-antigen is a strategy Enterobacteriaceae employ to escape the host immune system [24]. Based on the SPR experiments, we can conclude that AGP displays the strongest affinity for the *K. pneumonia* and *P. aeruginosa O*-antigen chemotypes (Figure 3). The *E.coli* O111:B4 and O127:B8 LPS chemotypes displayed comparable affinity, whereas the *S. enterica* and *S. marcescens *chemotypes displayed the lowest AGP affinity (Figure 3). In view of the variability of the *O*-antigen repeats across the test samples in terms of their sugar composition, acidic or neutral character, anomeric sugar linkage configuration, and branched structure (Table 1), we could not draw any correlations with these *O*-antigen properties and the AGP binding affinity of the LPS samples. Firstly, this finding would point out the importance of the unique composition of the *O*-antigen region for recognition by AGP, and secondly it would suggest that the ultrastructure and highly variable chain length of the *O*-antigen might play a larger role for the recognition of LPS by AGP.

### 4.2. The Significance of LPS-AGP Complexation *In Vivo*

The excessive release of inflammatory mediators in response to circulating LPS during Gram-negative bacterial infection can produce septic shock and multiple organ failure [2, 8]. When LPS circulates in the bloodstream and initiates endotoxemia, its first interactions are with the protein and cellular elements of blood. Plasma proteins that are known to interact with LPS include human serum albumin [53], immunoglobulin G [54], high-density lipoprotein (HDL) particles [55, 56], apolipoprotein A-II [57], LBP [58, 59], bactericidal/permeability-increasing protein (BPI) [58, 59], lactoferrin [60], hemoglobin [61], and AGP [39]. The early recognition of LPS by these factors is crucial for a number of protective host mechanisms to infection including catalyzing LPS transfer to TLR-4; restricting the spread of LPS from the site of infection via the bloodstream; directing LPS for clearance; binding and neutralization of the endotoxic lipid A component; and the eventual resolution of LPS-triggered inflammation [2, 3]. Our biophysical data underscore the observations that the endotoxic activity of LPS is subject to modification by binding to AGP [39].

The nanomolar LPS binding affinity of the LPS-specific plasma binding proteins, LBP and BPI, is significantly greater compared to the high micromolar affinity of AGP [58, 59]. Notwithstanding, the plasma concentrations of LBP (5–15 *μ*g/L) and BPI (<0.5 ng/mL) are much lower compared to that of AGP (0.7–1.0 g/L) [58, 59]. During infection, the plasma levels of LBP, BPI and AGP can increase up to 5-fold [13, 15, 35–37]. This situation would still present much more AGP binding sites for the binding of LPS compared to LBP and BPI. Therefore, despite the low affinity nature of the LPS-AGP interaction, the abundance of AGP in plasma, particularly as a result of the elevated levels during sepsis, presents sufficient *apo*-AGP sites for binding to LPS. Moreover, AGP appears to be more selective for specific *O*-antigen chemotypes of LPS (Figure 3), which contrasts the relatively nonspecific LPS recognition mechanism employed by LBP and BPI that is afforded by their selectivity for the conserved lipid A region of LPS [58], as opposed to AGP which appears to predominately participate in interactions with the highly variable *O*-antigen polysaccharide. 

### 4.3. Structure-Recognition Characteristics of the LPS-AGP Complex

We have employed the available crystallographic structure of the F1*S variant of human AGP to construct a model of the complex with Kdo2-lipid A (Figure 4). The model revealed some resemblances between the LPS binding mechanism of AGP and that of the polymyxin antimicrobial peptides [62]. Biophysical studies have shown that polymyxins bind to the disphosphoryl lipid A component of LPS firstly through charge and polar attractive forces and then through hydrophobic forces to the fatty acyl chains of the lipid A, leading to disaggregation of LPS [62]. The model suggests that similar to the LPS-polymyxin complex, the LPS-AGP complex is stabilized by both hydrophobic and polar interactions. The hydrophobic interactions involve the fatty acyl chains of lipid A and side chains in the ligand binding cavity of AGP. AGP is about 48% carbohydrate by weight, which would mean a significant area of the AGP surface that is available for intermolecular interactions is covered by *N*-glycans. In line with the SPR data, the model also suggests that polar interactions between the core and *O*-antigen of LPS with the surface *N*-glycans of AGP are involved in stabilizing the LPS-AGP complex. It is tempting to speculate that the formation of the LPS-AGP complex involves a two-step mechanism wherein the association initially relies upon a polar attraction between the core and *O*-antigen carbohydrate structures with the *N*-glycans that are proximal to the entrance of the AGP binding cavity. Once the polar attraction has been established, the fatty acyl chains of lipid A are able to insert into the nonpolar AGP cavity. This putative mechanism would be coincident with the weak affinity of AGP for diphosphoryl lipid A embedded in SUVs where the fatty acyl chains are inaccessible. 

The binding of AGP to LPS has previously been shown to be associated with the neutralization of the large negative electrophoretic mobility of AGP [39]. This would suggest that the surface charge distribution of AGP is altered upon LPS complexation, which is in agreement with the core/*O*-antigen-AGP *N*-glycan interactions inferred from our SAR data.

The reported cocrystallographic complex of chlorpromazine bound to the A variant of human AGP revealed that the chlorpromazine molecule largely occupies the central hydrophobic lobe I region of the AGP cavity [16]. The ability of chlorpromazine to displace FITC-LPS from AGP suggests that LPS competes for the same AGP binding site that is occupied by the chlorpromazine molecule. Coincidently, our model suggests that the *d*, R_2_, and *c* fatty acyl chains of lipid A occupy the lobe I region (Figure 4(b)). 

To further our understanding of the structure-recognition relationships of the LPS-AGP complex, it is important to understand the ultra-structure and composition of the interacting components under the solution conditions used for this study. Owing to its amphipathic nature, the LPS molecule normally exists as aggregates, as opposed to the mono-molecular form [9]. Bivalent cations (Ca^2+^ or Mg^2+^) are required for the dense packing of LPS within the outer membrane and when in an aggregated state [9]. The sequestration of these bivalent cations by chelating agents such as EDTA leads to the disruption of the intermolecular interactions between adjacent LPS molecules [9]. There were noticeable differences in the SPR interaction responses between AGP and some of the LPS samples in the presence and absence of EDTA (Figure 3), suggesting that the binding interaction is influenced by the ultrastructural organization of the LPS aggregates. In the case of the *E. coli* LPS samples, the binding responses were greater in the presence of EDTA, which decreases the aggregation state of LPS (Figure 3) [9]. In comparison, the binding responses with the LPS samples from the other strains was unaffected by the presence of EDTA in the SPR binding buffer. These differences would suggest that the binding of LPS by AGP may involve a complex series of interfacial molecular events in which the mono-molecular LPS is sequestered from the aggregate by AGP. Binding to LPS aggregates has been reported for other plasma proteins including lactoferrin [63], apolipoprotein A-II [64], HDL [65], hemoglobin [61], and the antimicrobial peptide polymxin B [62, 66]. 

The commercial AGP preparations derived from human plasma that we have employed throughout this study consist of proportions of the F1, S, and A variants in a nearly constant ratio of 40 : 30 : 30 (F1 : S : A) and are not desialylated [14, 49, 50]. In a previous study it was reported that *asialo*-AGP potentiated LPS-induced secretion of interleukin-1 *β*, interleukin-6, and tumor necrosis factor-*α* by human monocytes and macrophages to the same level as did native AGP [67]. This finding is inconsistent with the key role of the surface *N*-glycans on AGP for binding to LPS inferred from our biophysical data. 

### 4.4. Conclusions and Therapeutic Potential

The overtly promiscuous ligand binding cavity of AGP allows it to interact with generic structural components of many molecules which include exogenous inflammatory mediators such as LPS that are commonly circulating remnants of dead bacteria during infection. Based on available evidence, it has been proposed that AGP serves a protective role during infection by directly binding to LPS, neutralizing its direct toxicity and thereby downregulating the inflammatory response [39]. In this paper we have examined the interaction of LPS with AGP at a molecular level and studied the SAR. The *O*-antigen polysaccharide was found to be the key structure on the LPS molecule responsible for the recognition of LPS by AGP. The species and strain-specific variations in the *O*-antigen structure greatly affected the LPS binding affinity for AGP. Moreover, we present data that demonstrates that AGP protects against LPS-induced cytotoxicity *in vitro *(Figure 5). Our data is broadly consistent with the proposed role of AGP as an essential component in nonspecific resistance to Gram-negative infections [39]. If our findings are considered in terms of potential therapeutic applications, given that AGP is well tolerated [40], AGP could theoretically be administered prophylactically prior to large bowel surgery or during the acute stages of sepsis. 

## Supplementary Material

The raw SPR sensogram data for the AGP-LPS titrations is documented in Figure S1, available on-line.

## Figures and Tables

**Table 1:** The structures of the core oligosaccharide and *O*-antigen polysaccharides of the LPS samples used in this study. The general structure of the *Salmonella* LPS is shown in the first row. The stages at which the biosynthetic enzyme defects disrupt biosynthesis resulting in the production of the truncated “rough” LPS chemotypes R_a_ → R_e_ are indicated by the segmented arrows.

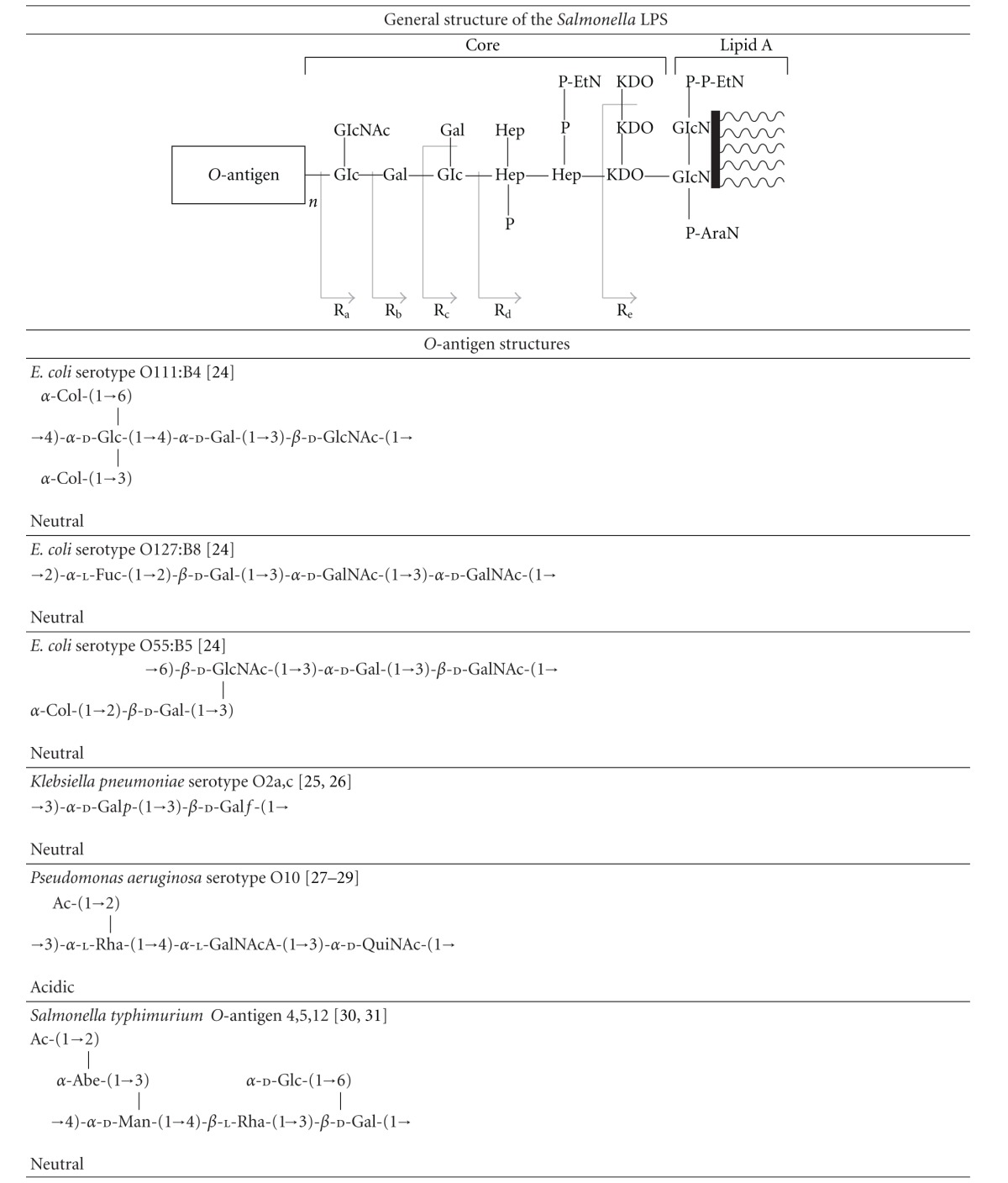 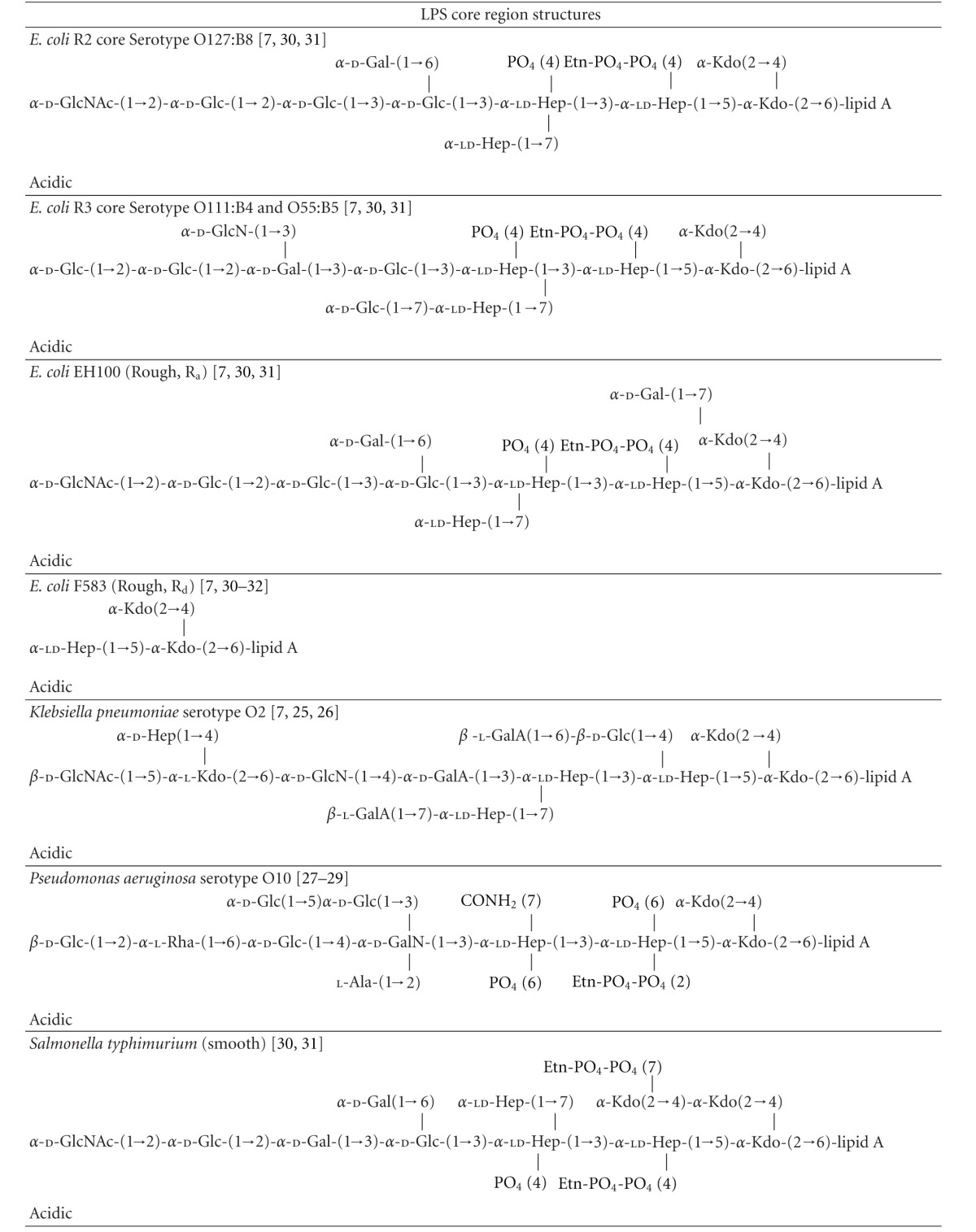 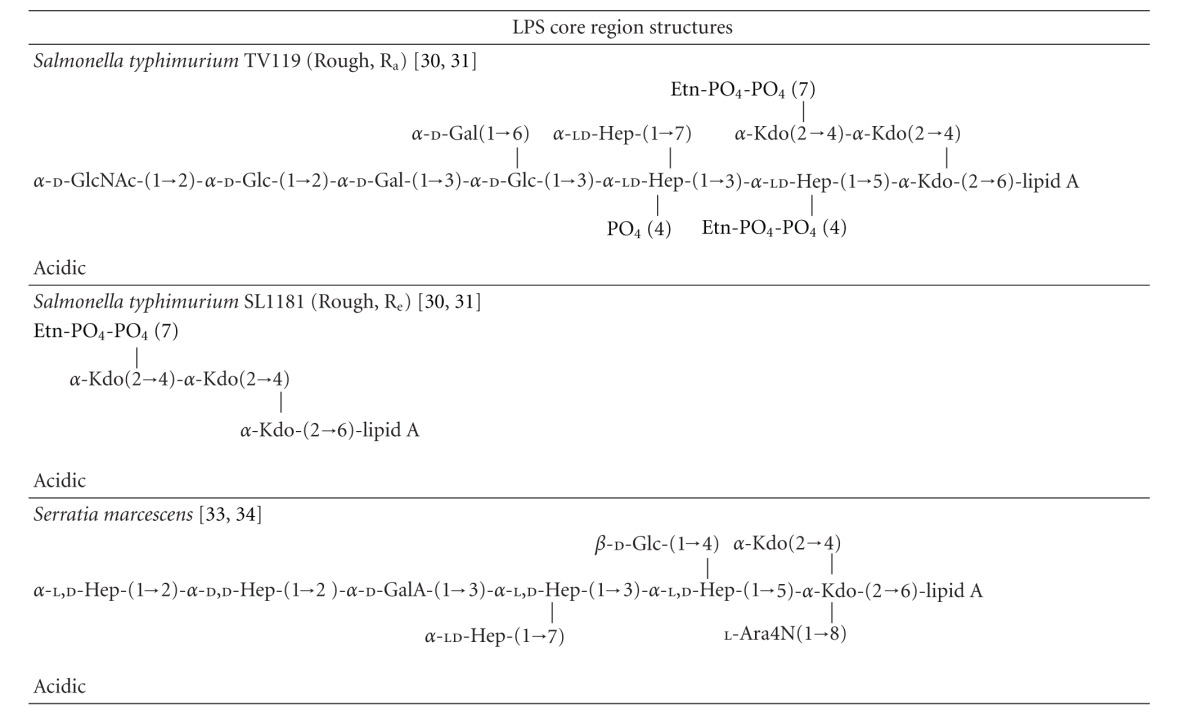

## Abbreviations

AGP: Human *α*-1-acid glycoprotein
FITC: Fluorescein isothiocyanate
ITC: Isothermal titration calorimetry
LPS: Lipopolysaccharide
LBP: Lipopolysaccharide-binding protein
Kdo: 3-deoxy-d-manno-oct-2-ulopyranosonic acid
OM: Outer-membrane
SPR: Surface plasmon resonance
SAR: Structure activity relationships
SUV: Small unilamellar vesicles
TLR4: Toll-like receptor 4.
