# Supplementary material for: Molecular Characterization of Lipopolysaccharide Binding to Human α-1-Acid Glycoprotein
Source: J Lipids. 2012 Dec 20;2012:475153. doi: 10.1155/2012/475153 (PMC3539403; doi:10.1155/2012/475153)
Supplement: Supplementary file 1 — The raw SPR sensogram data for the AGP-LPS titrations is documented in Figure S1, available on-line. [file 475153.f1.pdf]

Figure S1

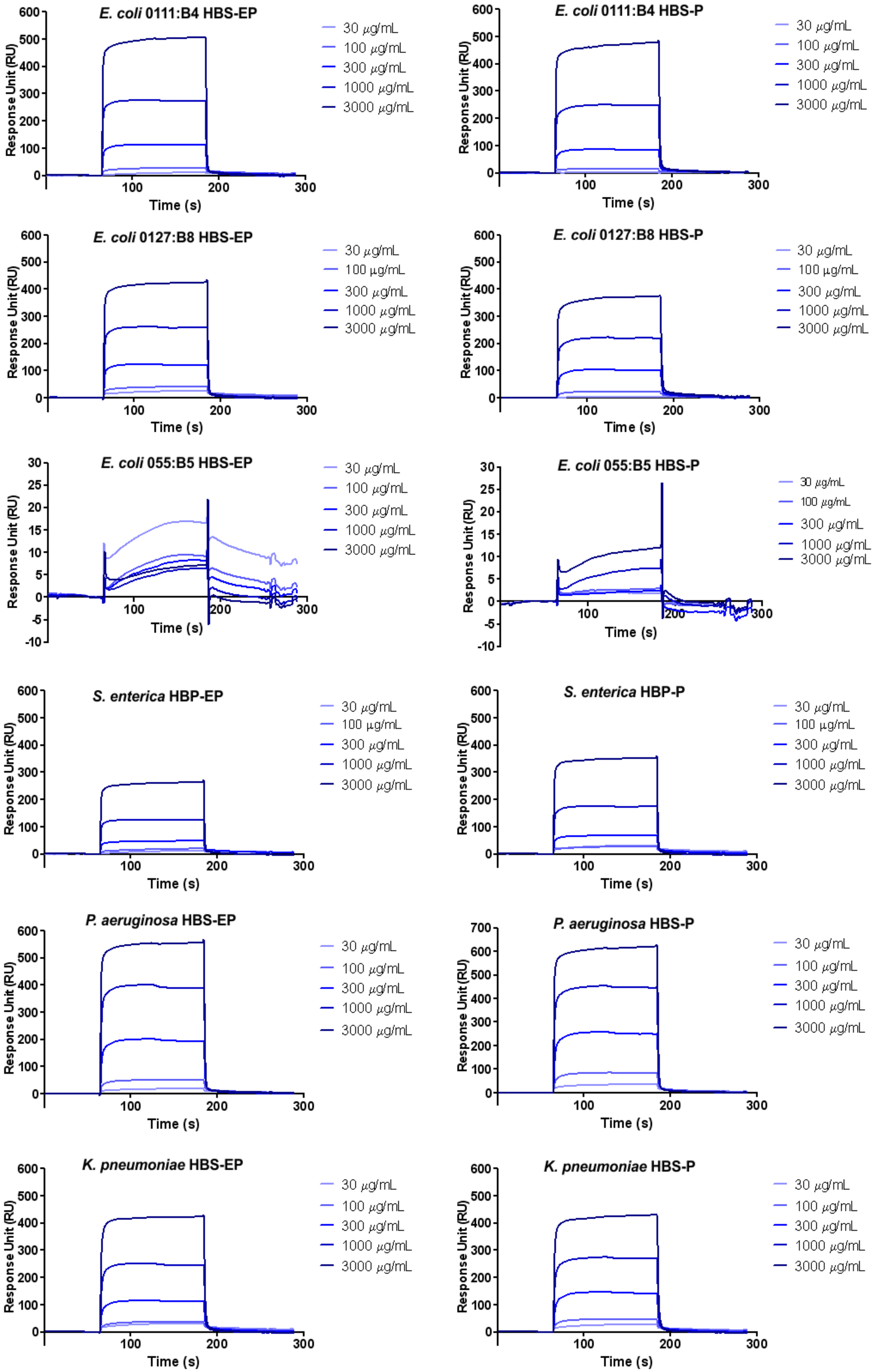
E. coli 0111:B4 HBS-EP
E. coli 0111:B4 HBS-P
E. coli 0127:B8 HBS-EP
E. coli 0127:B8 HBS-P
E. coli 055:B5 HBS-EP
E. coli 055:B5 HBS-P
S. enterica HBP-EP
S. enterica HBP-P
P. aeruginosa HBS-EP
P. aeruginosa HBS-P
K. pneumoniae HBS-EP
K. pneumoniae HBS-P
Response Unit (RU)
Time (s)
30 μg/mL
100 μg/mL
300 μg/mL
1000 μg/mL
3000 μg/mL

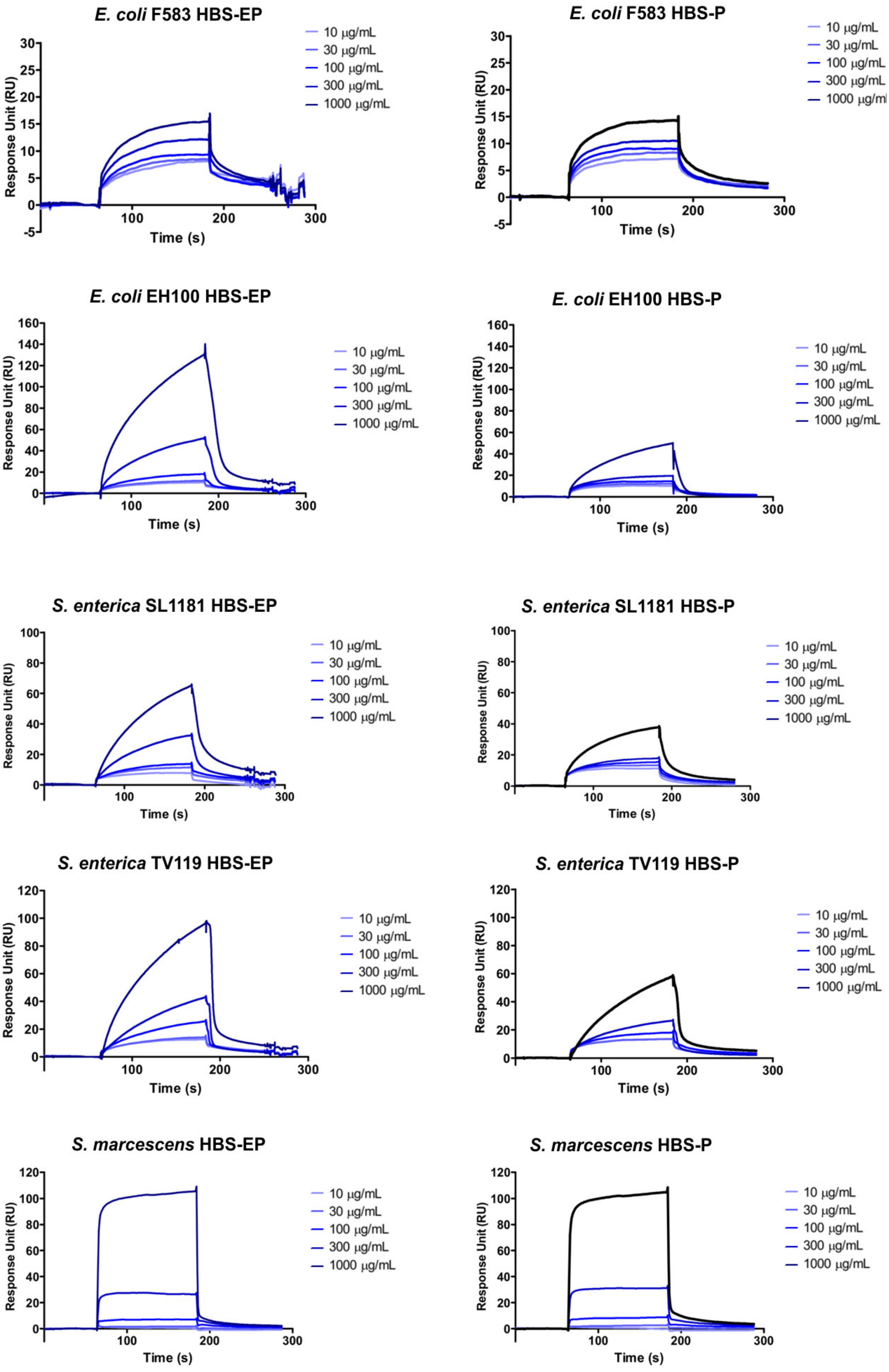
E. coli F583 HBS-EP
E. coli F583 HBS-P
E. coli EH100 HBS-EP
E. coli EH100 HBS-P
S. enterica SL1181 HBS-EP
S. enterica SL1181 HBS-P
S. enterica TV119 HBS-EP
S. enterica TV119 HBS-P
S. marcescens HBS-EP
S. marcescens HBS-P
Response Unit (RU)
Time (s)
10 μg/mL
30 μg/mL
100 μg/mL
300 μg/mL
1000 μg/mL
